# Radiation Dosimetry of Inhaled Radioactive Aerosols: CFPD and MCNP Transport Simulations of Radionuclides in the Lung

**DOI:** 10.1038/s41598-019-54040-1

**Published:** 2019-11-25

**Authors:** Khaled Talaat, Jinxiang Xi, Phoenix Baldez, Adam Hecht

**Affiliations:** 1 Department of Nuclear Engineering, University of New Mexico, Albuquerque, NM 87131 USA; 2 Department of Mechanical and Biomedical Engineering, California Baptist University, Riverside, CA 92504 USA; 3 Department of Biomedical Engineering, University of Massachusetts, Lowell, MA 01854 USA

**Keywords:** Risk factors, Biomedical engineering

## Abstract

Despite extensive efforts in studying radioactive aerosols, including the transmission of radionuclides in different chemical matrices throughout the body, the internal organ-specific radiation dose due to inhaled radioactive aerosols has largely relied on experimental deposition data and simplified human phantoms. Computational fluid-particle dynamics (CFPD) has proven to be a reliable tool in characterizing aerosol transport in the upper airways, while Monte Carlo based radiation codes allow accurate simulation of radiation transport. The objective of this study is to numerically assess the radiation dosimetry due to particles decaying in the respiratory tract from environmental radioactive exposures by coupling CFPD with Monte Carlo N-Particle code, version 6 (MCNP6). A physiologically realistic mouth-lung model extending to the bifurcation generation G9 was used to simulate airflow and particle transport within the respiratory tract. Polydisperse aerosols with different distributions were considered, and deposition distribution of the inhaled aerosols on the internal airway walls was quantified. The deposition mapping of radioactive aerosols was then registered to the respiratory tract of an image-based whole-body adult male model (VIP-Man) to simulate radiation transport and energy deposition. Computer codes were developed for geometry visualization, spatial normalization, and source card definition in MCNP6. Spatial distributions of internal radiation dosimetry were compared for different radionuclides (^131^I, ^134,137^Cs, ^90^Sr-^90^Y, ^103^Ru and ^239,240^Pu) in terms of the radiation fluence, energy deposition density, and dose per decay.

## Introduction

Radioactive aerosols arise from various sources such as nuclear accidents, natural decay processes, and the decommissioning of nuclear reactors^1–3^. Highly radioactive micro-particles were released to the surrounding environment in the Chernobyl and Fukushima Daiichi accidents^1,4^. The aerosol particles released from accidents and natural decay processes may remain suspended in the air for extended periods, be incorporated into soil particles that can reenter the air, or be inhaled by humans or animals. When aerosol particles are inhaled, a fraction of the inhaled particles deposit in the respiratory tract, while the rest is exhaled out^5–9^. The deposition fraction is dependent on many parameters such as the geometry of the respiratory airways, the particle size of the inhaled aerosol, and the breathing condition^10,11^. Inhaled radioactive aerosols can expose internal organs to radiation for extended periods and may induce a spectrum of functional and morphological changes, such as genetic mutations and carcinogenesis^12,13^. Their severity can be related to the absorbed radiation dose in the different specific tissues, which is further related to the number of radioactive particles inhaled, how long they stay, and the type of radiation they emit. Unlike fat and muscles being the main internal defense to external exposures, there is nothing to protect cells and tissues of susceptible organs from the internal harming effects. Once inhaled into the lungs, much of the radiation energy will get absorbed by cells, tissues, and organs. The association of radioactive exposure and lung cancer has been well documented in populations exposed to residential radon^14,15^, children in nuclear-contaminated areas^16^, Mayak workers^17,18^, fluorspar miners^19^, and workers in Uranium mines^20,21^, mills^22,23^, or enrichment^24^. The correlation between lung carcinoma and internally deposited radionuclides were reviewed by Bair^25^, Harrison and Stather^26^, Harrison and Muirhrad^27^, and Raabe^28^. It should be noted, however, that radioactive exposures from particulates even while they are in the lung can affect the organism as a whole and not the lung alone as a peripheral dose can be delivered to nearby organs. The biological pathways from the respiratory tract to the rest of the body depend on the dissolution of particles in the lung fluids and absorption into the blood and then transfer to other organs. This is very specific to the chemical matrix of the particle and is commonly treated with a compartment model with different transfer rates^29^. Even though the respiratory tract has been examined with compartment models^30,31^, fine details on particulate distributions within the respiratory tract were neglected and treated as evenly mapped within each compartment. Moreover, the geometrical fineness of the respiratory tract was often simplified in the compartment models, which consisted of a limited number of regions without anatomical details. These can range from three-compartment models^14^ to ten-compartment models^13^.

In the present work, computational fluid-particle dynamics (CFPD) is used to understand the probabilities of particle deposition in different regions of the respiratory tract with a geometrical fineness beyond that of the compartment model. CFPD maintains knowledge of the size and location of the deposited particles throughout the geometry, which is essential for understanding therapeutic outcomes, pollution irritation, and other lung effects. The approach of using CFPD for aerosol deposition in image-based human airways has demonstrated success in pharmaceutical research with flexibility and control over respiratory conditions and particle sizes but has not been widely used in radiation dosimetry. The granular information on particle size and position can also be used as a starting kernel for radiation simulations within the lungs, as is performed here, or for follow-on biokinetics studies^29^. Dissolution and absorption to transport throughout the body is beyond the immediate scope of this work. There are many cases in which radionuclides have a long residence time within the lungs for which this work can be directly applied, for example, Newton^32^ shows a ^239^PuO_2_ lung clearance with a biological half-life of ∼800 days.

For radiation modeling, the Monte Carlo (MC) method has been widely used in radiation studies since the 1970s. It stochastically simulates radiation transport and their interactions with surrounding matter such as gamma-ray absorption and scattering. MC applications in medical physics range from calculating fundamental dosimetry variables to simulating radiotherapy treatment planning. However, it was only since the 1990s that MC developments have been made for the direct simulation of dose distributions within a patient using phase-space data impinging on 3D CT images^33^. The Los Alamos Monte Carlo N-Particle transport code version 6 (MCNP6) provides accurate consideration of fundamental particle interactions with matter, supports sophisticated geometry models, and allows for simulations of the photon/electron transport energy below the conventional 1-kilovolt limit^34,35^. MCNP has been demonstrated to be sufficiently accurate in a wide range of radiation applications, such as metal cytotoxic effects^36^, medical linear accelerator^37^, neutron biological effects^38^, plutonium content verification^39^, neutron detector design^40^, and radiotherapy treatment planning^41,42^.

**Figure 1:**
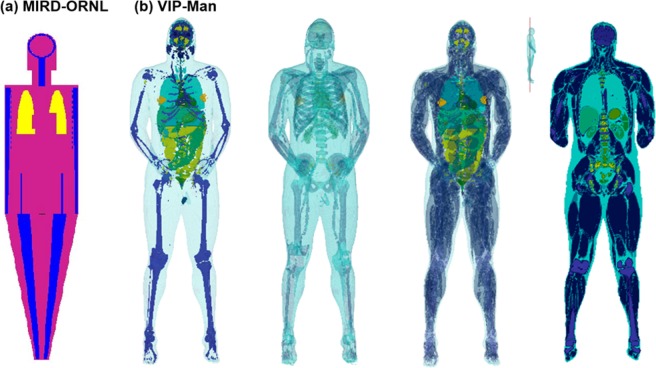
Computational phantoms for radiation dosimetry: (**a**) Stylized MIRD-ORNL model and (**b**) Image-based VIP-Man model with different organs turned on (left four panels).

Computational phantoms for radiation dosimetry have evolved significantly in the past five decades^43–45^. Stylized phantoms that were based on simple quadratic equations such as MIRD family phantoms^46,47^, ADAM and EVA^48^, and KMIRD^49^ were developed and widely used since the 1960s. The key advantage of the stylized phantoms was that they weren’t computationally demanding and didn’t represent a specific individual. However, they lacked anatomical detail and accurate physiological representation. With the advent of computed tomography (CT), voxel models such as Zubal^50^, VIP-Man^51^, NORMAN^52^, KTMAN^53^, CNMAN^54^, and ICRP reference phantoms^55^ were developed and widely used in nuclear medicine. A comparison between MIRD-ORNL phantom and VIP-Man phantom is shown in Fig. 1. The advantage of voxel phantoms over stylized phantoms was the increased accuracy in anatomical detail and physiological representation, as shown in the figure. However, voxel-based phantoms are difficult to deform and manipulate, which inspired the development of BREP and NURBS based phantoms such as the 4D VIP-Man chest^56^ and RPI-Pregnant females^57^. Much of the computational modeling work on radioactive aerosol dosimetry was done in the 1970s and later in the years following the Chernobyl accident using stylized MIRD-type phantoms and compartment respiratory tract models^58,59^. Modern developments in computational fluid dynamics have enabled the simulation of aerosol transport in human airways^10,60–62^. Combined with Monte Carlo radiation transport and voxel-based models, a physically informed model of radiation transport due to radioactive aerosols in the respiratory tract can be developed potentially allowing for more accurate and more robust assessment of the internal dose distribution than the traditional approach which heavily relied on experimental data and simplified airway models^63^. Additionally, voxel phantoms such as the VIP-Man model, which has ~6 million voxels, have significantly more sophisticated geometries than constructive solid geometry based MIRD model and other simple stylized phantoms. As a result, there is a need to develop systematic tools to determine the internal source distribution after exposure. To this aim, computational fluid-particle dynamics (CFPD) simulations combined with computer codes for particle registration and visualization could provide that spatial source distribution information to Monte Carlo radiation transport models.

**Figure 2:**
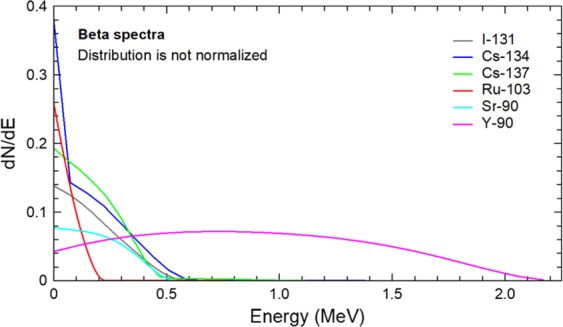
Beta spectra for ^131^I, ^134,137^Cs, ^103^Ru, and ^90^Sr-^90^Y.

Upon being inhaled into the lungs, the deposition rate and locations of radionuclides are highly sensitive to the diameter of their carrier aerosols, which are generally heterogeneous in size^61,64^. Particles smaller than 0.5 μm can penetrate into the alveoli and deposit there, even though their deposition patterns may still be size dependent^10,62,65^. Large particles don’t closely follow the flow and tend to deposit earlier in the upper airways. Previous studies have demonstrated that particles larger than 10 μm tend to collect in the nose or mouth, while particles in the range 4–6 μm deposit in the pharynx region^7,66,67^. To accurately estimate the aerosol deposition and distribution, it is crucial to identify the particle size range for radioactive aerosols of interest. In this work, we examine aerosols from the Chernobyl and Fukushima accidents, and Table 1 summarizes the properties of several different aerosols that arise from these accidents^68–71^. The activity median aerodynamic diameter (AMAD) data in Table 1 is compiled from multiple sources. The geometric standard deviations (GSD) are also presented. It should be noted that measurements of AMAD are scattered in the literature and are not unanimous as the size distribution varies depending on the geographical location where the samples were obtained, altitude, and time after release^70,72,73^. The AMAD values presented in Table 1 are used in the simulations in order to demonstrate the method with realistic particle sizes but should not be considered universally characteristic of the particular radionuclides. In the particular case of ^90^Sr and ^90^Y which are strong beta emitters, the simulations were conducted using 0.42 µm AMAD and GSD of 3.5, similar to I and Cs values, due to the lack of data on their AMAD from air samples in Chernobyl and Fukushima accidents. The decay modes, different radiation yields and energies, and half-life data were obtained from NUDAT2.7^74^. Beta spectra for different radionuclides considered in this work are shown in Fig. 2 based on data from the IAEA chart of nuclides^75^. It has been demonstrated that using average energies instead of a spectrum can overestimate the local deposition of the radiation in tissue particularly for radionuclides that emit energetic betas such as ^90^Y as the averages tend to be shifted to lower energies which have a shorter mean free path in tissue^76^. Fig. 2 shows that ^90^Y, which results from ^90^Sr decay emits energetic betas in comparison with other radionuclides, while ^103^Ru tends to emit lower energy betas. As a result, the energy deposition for ^103^Ru due to beta radiation is expected to be more localized in the lung and trachea, while that of ^90^Y may reach other nearby organs such as the heart and liver. The present simulations used beta spectra rather than averages. The normalization of the beta spectra is not relevant herein as MCNP6 normalizes the distribution on its own.

**Table 1:** Properties of Some Radioactive Aerosols of Interest.

Radioactive Isotope	Reported Aerosol Particle Size	Primary Radiation	Gamma Energy (keV)	Gamma Yield (%)	X-Ray Energy (keV)	X-Ray Yield (%)	Half-Life
^131^I	AMAD: 0.42 μm GSD: 3.5^70^	β- & γ	80.2	2.62	4.1	0.63	8.052 d
			85.9	0.00009	29.5	1.53	
			163.9	0.0211	29.8	2.82	
			177.2	0.269	33.6	0.26	
			232.2	0.0032	33.6	0.51	
			272.5	0.0576	34.4	0.15	
			284.3	6.12			
			295.8	0.0018			
			302.4	0.0047			
			318.1	0.0774			
			324.7	0.0212			
			325.8	0.273			
			358.4	0.016			
			364.5	81.5			
			404.8	0.0546			
			449.6	0.0073			
			503	0.359			
			637	7.16			
			642.7	0.217			
			722.9	1.77			
^134^Cs	AMAD: 0.43 μm GSD: 3.6^70^	β- & γ	127.5	12.6	4.5	0.105	2.065 y
			138.7	0.004	11.2	1.08	
			232.6	0.001	31.8	0.238	
			242.7	0.027	32.2	0.434	
			326.6	0.016	36.3	0.042	
			475.4	1.477	36.4	0.08	
			563.2	8.338	37.3	0.025	
			569.3	15.37			
			604.7	97.62			
			795.9	85.46			
			802	8.688			
			847	0.0003			
			1038.6	0.99			
			1168	1.79			
			1365.2	3.017			
^137^Cs	AMAD: 0.43 μm GSD: 3.7^70^	β- & γ	283.5	0.00058	4.5	0.91	30.08 y
			661.66	85.1	31.8	1.99	
					32.2	3.64	
					36.3	0.35	
					36.4	0.67	
					37.3	0.21	
^103^Ru	AMAD: 0.83 μm GSD: 1.5^73^	β- & γ	39.8	0.069	2.7	4.03	39.26 d
			42.6	0.005	20.1	2.49	
			53.3	0.443	20.2	4.73	
			62.4	0.0004	22.7	0.393	
			113.2	0.003	22.7	0.76	
			114.9	0.007	23.2	0.182	
			241.9	0.014			
			292.7	0.001			
			295	0.288			
			317.7	0.009			
			357.4	0.009			
			443.8	0.339			
			497.1	91			
			514.4	0.006			
			557.1	0.841			
			567.7	0.002			
			610.3	5.76			
			612.1	0.105			
			651.7	0.0002			
^239/240^Pu	AMAD: 2 μm GSD: 1.8^71^	α ~ 5.15 MeV	NG*	NG	NG	NG	24100 y
							6560 y

NG: negligible dose compared to main decay mode < 0.5% in total.

The objective of this study is to evaluate the organ-specific radiation dosimetry from inhaled radioactive aerosols that are in the respiratory tract by integrating the CFPD and MCNP codes. Specific aims include

1. To simulate the deposition rate and mapping in a physiologically accurate mouth-lung model (Fig. 3) of inhaled radioactive aerosols with heterogeneous size distributions.

**Figure 3:**
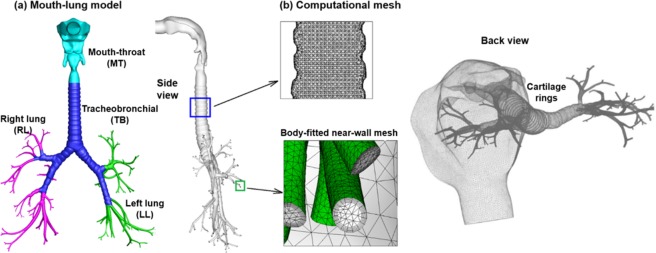
Mouth-to-lung model: (**a**) surface geometry and (**b**) computational mesh. Cartilage rings were retained in the tracheobronchial region.
2. To couple the CFPD-predicted deposition data from ANSYS FLUENT with MCNP transport code and VIP-Man whole-body voxel phantom.
3. To simulate the spatial distribution of energy deposition density for different radionuclides: ^131^I, ^134,137^Cs, ^90^Sr-^90^Y, ^103^Ru and ^239,240^Pu and estimate short-term organ doses due to radiation emitted from the lung when these radionuclides are inhaled.

The newly developed CFPD-MCNP model considers anatomical details of the respiratory tract, a whole breathing cycle, polydisperse aerosol distributions, and heterogeneous deposition pattern. This study does not include the biokinetics of transport of the radionuclides to other organs, as that is specific to the containing aerosol particle, but serves as the starting point for such studies. As a consequence, the dose distributions calculated in the present work represent the case when aerosols are freshly inhaled but may not represent the cumulative dose distribution.

## Results

### Airflow

**Figure 4:**
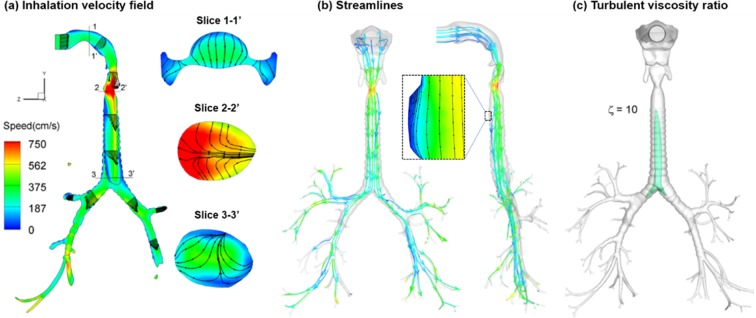
Inspiration air flows: (**a**) inhalation velocity field, (**b**) streamlines, and (**c**) turbulent viscosity ratio, ξ.

Detailed knowledge of respiratory airflow is the first vital step to accurately predict the behavior and fate of inhaled radioactive aerosols. The velocity field and stream traces in the mouth-lung geometry are illustrated in Fig. 4 as sagittal and coronal contour profiles. In Fig. 4a, skewed velocity profiles are noted in the upper airway, with the main flow shifted to the dorsal wall of the pharynx. In the larynx, the airflow accelerates prior to the glottis due to the gradual airway narrowing. A laryngeal jet forms at the glottis and moves along the upper trachea (Fig. 4b). Due to the airway expansion downstream of the glottis, a large recirculation zone develops, which displaces the flow jet and cause it to oscillate. This jet instability is referred to as the Coanda effect and has been frequently reported in previous experiments of the larynx^77–79^. In the cartilaginous rings, smaller eddies are noted (Fig. 4b). To illustrate the secondary motions in these regions, 2-D velocity contours and streamlines are shown in selected coronal planes (1-1′–3-3′ in Fig. 4a). The magnitude of the secondary motion is about 25% of the main flow. It functions to mix the inhaled airflow and distribute the inhaled air towards the wall.

Highly heterogeneous flow features are observed in the TB region (Fig. 4a,b). The left-right asymmetry is obvious in the two main bronchi. In the model considered, airflows in nearly all branches are still developing, and haven’t reached a parabolic profile. This is different from certain previous studies that typically assumed developed flows in distal bronchioles after G6^80,81^. Moreover, the velocity profiles in the distal bronchioles can differ significantly from one another. Therefore, a single-branch lung model as proposed in Tian *et al*.^82^ may not adequately represent the whole lung scenario.

To examine turbulent features of the inhaled flow, the turbulence viscosity ratio, ζ is shown in Fig. 4c. Turbulent flow can significantly affect aerosol transport and deposition. From Fig. 4c, turbulence occurs mainly downstream of the glottis. The turbulence peak inside the trachea may be attributed to both laryngeal jet effects and cartilaginous perturbations. The oral cavity and distal bronchioles are still dominated by laminar flows.

### Aerosol deposition

**Figure 5:**
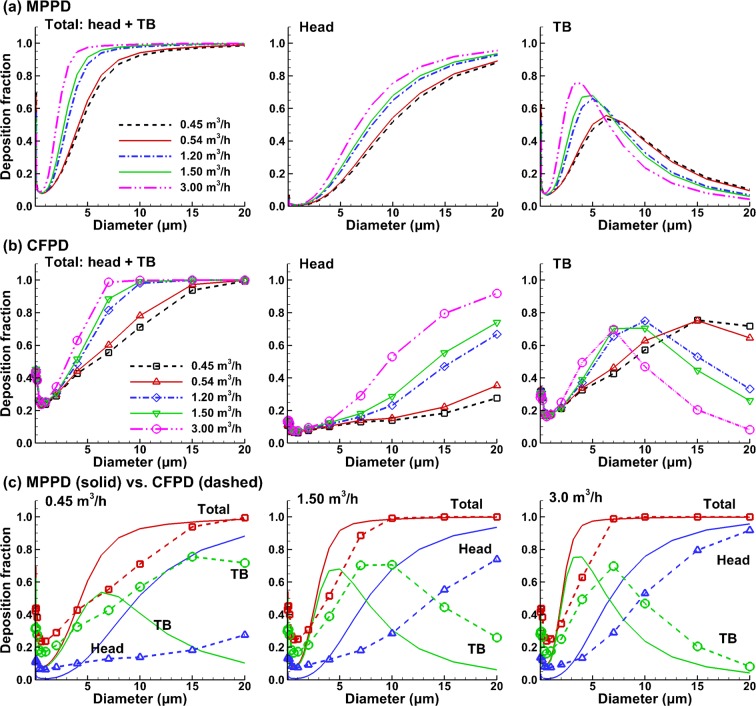
Comparison of the deposition fractions between the CFPD and MPPD models with oral breathing: (**a**) MPPD predictions in the head and TB (total and regional) under five breathing conditions, (**b**) corresponding CFPD predictions, and (**c**) comparison between MPPD and CFPD predictions on the total and regional basis for 0.45 m^3^/h, 1.5 m^3^/h, and 3.0 m^3^/h.

Figure 5 shows the comparison of deposition fractions between the CFPD and MPPD (multiple-path particle dosimetry model) predictions under different breathing conditions. Explicit deposition data with mouth breathing haven’t been found in ICRP literature. As a result, the MPPD model, which was based on several models including the 1994 ICRP model, was selected to compare to the CFPD results of this study^8,83,84^. The MPPD-predicted dosimetry is shown in Fig. 5a in the head (mouth-throat) and tracheobronchial (TB) region on a total and regional basis. The respiratory flow rates of interest included 0.45 m^3^/h (sleeping), 0.54 m^3^/h (sitting), 1.5 m^3^/h (light working), 3.0 m^3^/h (heavy working), as well as a nominal ICRP flow rate of 1.2 m^3^/h that represented a mixture of 2.5-hour sitting (0.54 m^3^/h) and 5.5-hour light working (1.5 m^3^/h). The corresponding CFPD-predicted results are shown in Fig. 5b. In order to determine whether significant differences exist between the CFPD and MPPD (or ICRP) predictions in the mouth-breathing dosimetry, the same physiological conditions were ensured in the two models as much as possible. These include the same flow rates, deposition from both inhalation and exhalation, a comparable upper respiratory tract volume (53 ml), upright position, and no pause. The TB region represents the conducting airway from the trachea to G8 in both the CFPD and MPPD (as well as in ICRP) models^9^. The particle loss in the pulmonary region was also accounted for in the CFPD predictions when considering the expiratory particle deposition^83^, as detailed in the Methods section.

In general, a qualitative agreement in the dosimetry profiles was observed between the MPPD and CFPD results, despite the apparent disparities in their magnitudes (Fig. 5a,b). A rapid increase of the head deposition fraction (DF) with particle size and a modal profile in the TB deposition were observed in both MPPD and CFDP predictions. Moreover, the modal peak in the TB deposition shifts to the right with decreasing flow rates in both models. However, the flow-dependency of the deposition differs, which is more pronounced regionally than the total airway. In regions (head and TB) where notable difference exists, the amount of difference also varies with the particle size. In Fig. 5b (middle panel), the discrepancies between the head DFs are much larger than those in Fig. 5a, suggesting a higher sensitivity of the CFPD model to the flow rate and particle size than the MPPD model. Similarly, the shift amounts of the TB modal peaks are much larger in the CFPD model in comparison to the MPPD model (right panels, Figs. 5a vs. 6b).

A quantitative comparison between the CFPD and MPPD results is shown in Fig. 5c for three breathing scenarios: 0.45 m^3^/h (sleeping), 1.5 m^3^/h (light working), and 3.0 m^3^/h (heavy working). Overall, larger differences were observed in the regional (head and TB) than total deposition, and these differences are more obvious under lower physical activity conditions. It is interesting to note that, despite the huge DF disparities in the head and TB at 0.45 m^3^/h between CFPD and MPPD, their combined DFs are similar (left panel, Fig. 5c). Improved agreements were achieved at higher flow rates. In particular, relatively good agreement was observed between CFPD and MPPD at 3.0 m^3^/h in both the regional and combined DFs, with similar magnitudes in the asymptotical DF profiles (blue) in the head and the modal profiles in the TB (green), as displayed in the right panel of Fig. 5c.

**Figure 6:**
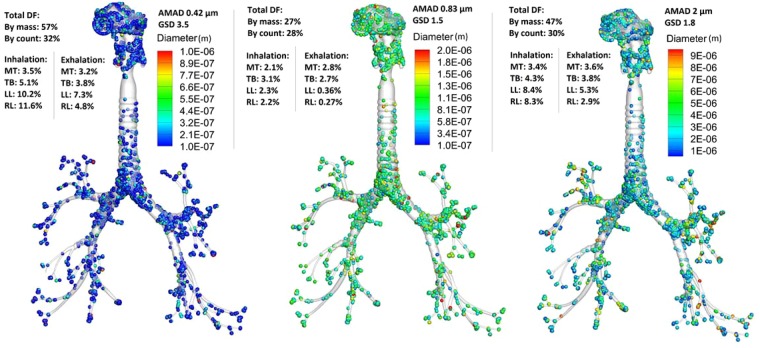
Deposition distribution of polydisperse aerosols with different particle size distributions: (**a**) ^131^I with AMAD of 0.42 µm and GSD of 3.5, (**b**) ^103^Ru with AMAD of 0.83 µm and GSD of 1.5, and (**c**) ^239,240^Pu with AMAD of 2.0 µm and GSD of 1.8.

For the high spatial resolution CFPD simulations, deposition of polydisperse aerosols with log-normal distributions are shown in Fig. 6 for three aerosol groups. It is noted that the deposition fractions were calculated on a mass basis (the ratio of deposited particle mass over inhaled mass) instead of the count basis that was conventionally adopted for monodisperse aerosols. The distribution with AMAD of 0.42 μm and GSD of 3.5 (I, Cs, Y-Sr) had the largest deposition fraction by mass of 57% compared to 27% in the 0.83 μm AMAD case with 1.5 GSD (Ru) and 47% in the 2 μm AMAD case with 1.8 GSD (Pu). For comparison purposes, the deposition fraction by particle count is also listed in Fig. 6, which is different from that by mass. Deposition rates in sub-regions of the airway are also listed in Fig. 6 for both inhalation and exhalation phases. During inhalation, 70–90% of the aerosols by mass passed through the upper airway and entered the alveoli. As modeling the alveoli is not computationally feasible due to millions of alveolar sacs with varying orientation, empirical correlations for acinar deposition were implemented. The alveolar-deposited aerosols were assumed to locate in the most distal bronchiole outlets. During exhalation, a fraction of particles was removed to account for alveolar deposition. Approximately 62–85% of the particles by mass were exhaled through the mouth opening (Fig. 6).

In contrast to monodisperse aerosols, deposition fractions of polydisperse aerosols are very sensitive to the presence of large-sized particles (outliers in distribution). Having even a small amount of micron particles can substantially skew the mass deposition fraction; this explains the apparent difference between the mass- and count-based deposition fractions for the 0.42 AMAD case that has the largest size range (GSD 3.5) among the three aerosol groups. To elaborate with an example, one outlier of 4.2 μm particle carries a mass of more than 1,000 times that of particles of the median diameter (0.42 μm).

**Figure 7:**
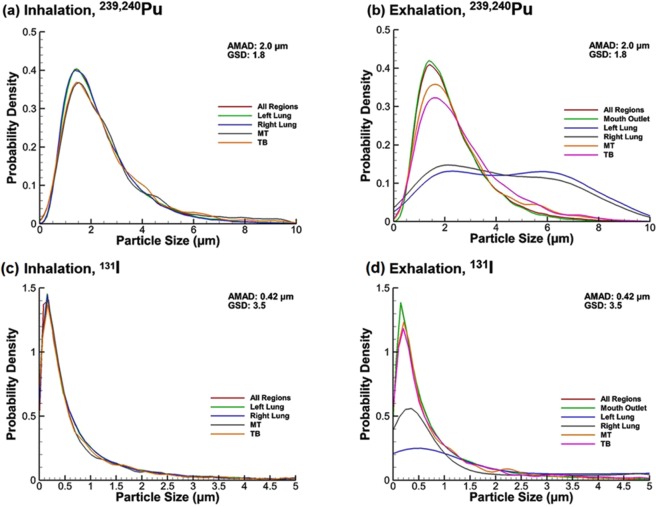
Particle size distribution in different regions of the respiratory tract for ^239,240^Pu at the end of (**a**) inhalation and (**b**) exhalation, and for ^131^I at the end of (**c**) inhalation and (**d**) exhalation, respectively.

Figure 7 shows the particle size distribution in different respiratory regions at the end of inhalation and exhalation for two aerosol groups. For both aerosols considered, for the inhalation phase, particle size distributions remain similar as the initial lognormal profile in different regions (Fig. 7a,c) By contrast, large differences of the aerosol profiles were captured during exhalation for both aerosols; these differences are most pronounced in the left and right lungs (Fig. 7b,d), presumably owing to the depletion of large micron aerosols.

### MCNP6 statistical relative error

**Figure 8:**
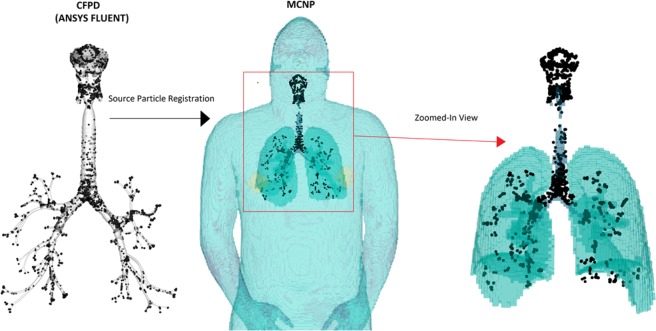
Registration of aerosol particles from CFPD predictions into the VIP-Man phantom as gamma source particles in MCNP. Aerosol sizes were scaled up to visualize the deposition locations relative to the lung phantom.

**Figure 9:**
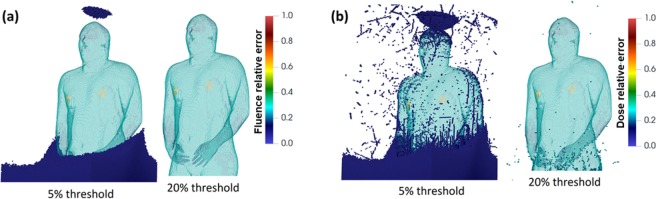
Statistical relative error in (**a**) fluence, and (**b**) energy deposition tally. Note: turquoise color is the skin and not relative error.

After obtaining the 3D deposition distribution using CFPD, aerosol particles on the airway surfaces was registered with the lung space in the VIP-Man phantom, as shown in Fig. 8. Details of the development of computer codes to visualize MCNP6 output, register source particles in MCNP6 based on CFPD results, consider bronchiolar and alveolar deposition, and calculate mass-based deposition fractions of polydisperse particles were documented in Methods. Figure 9 shows the statistical relative error for the radiation fluence and radiation energy deposition after the MCNP6 simulations were run for 100 million histories. The relative errors for gamma-ray dose in the torso were below 2%. As fewer photons passed the torso, the relative errors in regions away from the lungs, such as the legs, were higher (~20%). However, these regions are not of interest since the doses that they receive are orders of magnitude smaller than the organs in the torso. While each case requires a different number of histories depending on source location, energy, mesh tally size and the acceptable relative error, the choice of 100 million histories was guided by other studies that used the VIP-Man model and determined that 100 million histories were adequate to obtain statistically sound results for the cases that they modeled^85,86^.

### Photon/beta fluence per decay and energy deposition density

Gamma-ray and alpha-particle energies have discrete values for each decay transition, as presented in Table 1, while beta particles have a spectrum for each transition. Using just a single average energy would underestimate the peripheral dose to neighboring organs, which can be strongly dependent on the high energy tail of the distribution. The spectra for all beta emitters considered here are shown in Fig. 2, with the short-lived decay product of ^90^Sr, ^90^Y, having the highest endpoint energy of >2 MeV. Thus, fluence of some betas can be considered, not only gamma fluence.

**Figure 10:**
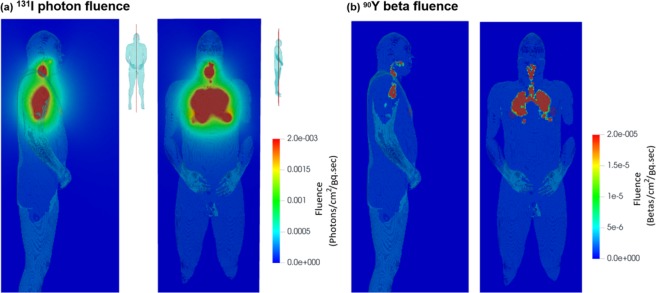
Cross-sectional view of (**a**) the gamma and x-ray fluence per decay from ^131^I, and (**b**) beta fluence per decay from the ^90^Y beta spectrum, both on linear scales.

Figure 10 shows the cross-sectional view of the photon fluence per decay for ^131^I due to gamma rays and x-rays, and ^90^Y due to beta particles. A 3D transparent body phantom was superimposed to illustrate the position of the photon fluence, calculated for photons and beta particles using the *fmesh* tally in MCNP6. Figure 10a displays the mid-sagittal and coronal photon fluence on a linear scale, which exhibits hot spots in both the throat and lung parenchyma (Fig. 10); this observation is consistent with the aerosol deposition distribution in Fig. 6 that the flow-limiting glottis and successively bifurcating bronchioles received the maximum aerosol doses. The photon fluence intensity decreases quickly as it moves away from the lungs (Fig. 10, left). In contrast, very localized fluence was observed in the respiratory tract for beta-emitting ^90^Y (Fig. 10, right). There are clear interfaces between the respiratory tract and surrounding tissues, indicating a short distance of travel for most beta radiation. It is noted that the fluence is dependent on both the source radiation and the properties of the medium that the photon or particle travels through. Relevant medium properties for photons and charged particles include atomic number, atom density, and composition, all of which can vary spatially and affect the interactions with the particles. In the VIP-Man phantom, the composition of the material varies spatially on an organ basis. Table 2 shows the tissue properties of some vital organs of interest in this work. Considering the high densities of the organs, it is expected that the fluence intensity in the legs is much lower than that of the surrounding air, as evident in Fig. 10.

**Table 2:** Tissue properties of vital organs in proximity to the radiation sources in the VIP-Man phantom.

Organ Name	Material Density (g/cc)	Material Composition (Mass Fractions)		
Lung and Respiratory System	0.26	H: 0.103	O: 0.749	S: 0.003
		C: 0.105	Na: 0.002	Cl: 0.003
		N: 0.031	P: 0.002	K: 0.002
Heart wall	1.03	H: 0.103	O: 0.734	S: 0.002
		C: 0.121	Na: 0.001	Cl: 0.003
		N: 0.032	P: 0.001	K: 0.002
				Fe: 0.001
Liver	1.05	H: 0.102	O: 0 .716	S: 0.003
		C: 0.139	Na: 0.002	Cl: 0.002
		N: 0.030	P: 0.003	K: 0.003
Thyroid	1.05	H: 0.104	O: 0.745	S: 0.001
		C: 0.119	Na: 0.002	Cl: 0.002
		N: 0.024	P: 0.001	I: 0.001
Bones	1.55	H: 0.034	O: 0.435	P: 0.103
		C: 0.155	Na: 0.001	S: 0.003
		N: 0.042	Mg: 0.002	Ca: 0.225
Muscles	1.04	H: 0.102	O: 0.710	S: 0.003
		C: 0.143	Na: 0.001	Cl: 0.001
		N: 0.034	P: 0.002	K: 0.004

**Figure 11:**
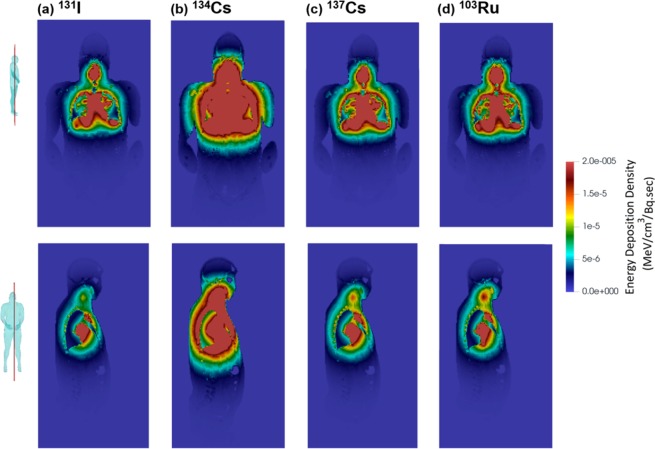
Comparison of energy deposition densities (MeV/cm^3^) on coronal (upper row) and sagittal (lower row) planes from gamma and x-rays from: (**a**) ^131^I, (**b**) ^134^Cs Cs-134, (**b**) ^137^Cs, and (**d**) ^103^Ru.

Figure 11 shows the spatial distribution of the energy deposition densities from different beta and gamma-/x- ray emitting radionuclides (^131^I, ^134^Cs, ^137^Cs, and ^103^Ru) within the respiratory tract. For gamma- and x-ray radiation, the energy deposition density is calculated by integrating the photon fluence with the atom density of the material, the total microscopic cross-section and the photon heating number over the space-momentum phase space in each mesh element using a tally modifier card in MCNP6^87^. The *fmesh* tally works with photons, and gives spatial fineness to photon energy deposition for visualization. Beta particle energy deposition density cannot be calculated the same way in MCNP6 but, like alpha particles, the energy deposition density can be calculated with *tmesh*, which does not give fluences. Due to the very local energy deposition, alpha particle fluence was not calculated. All particle contributions to organ doses are recorded. Overall, the high energy zones for the four radionuclides are confined in the upper torso from the mouth to slightly below the diaphragm (Fig. 11). It is noted that the blue man silhouette in each panel is not a superimposed model; it is the physical contour of energy distribution due to the different absorption levels in the brain, limbs, bones, and subcutaneous tissue.

Different energy deposition patterns are observed in Fig. 11 among the four radionuclides, with ^134^Cs (with a dominant 796 keV emission) exhibiting the highest energy density level while the other three exhibiting similar patterns. Further examination also reveals subtle differences among the three, with ^131^I having a lower energy density than the other two, especially in the throat (Fig. 11a *vs*. 11d). As noted before, the radionuclides considered (^131^I, ^134^Cs, ^137^Cs, and ^103^Ru) are different from each other not only in gamma-ray, x-ray, and beta-particle energies, but also in aerosol size distribution, deposition fraction, and deposition pattern.

### Radiation dose

**Figure 12:**
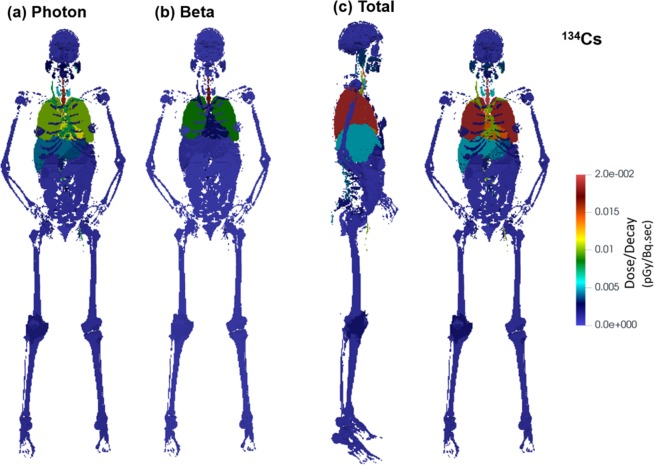
Dose to organs per decay from ^134^Cs in the respiratory tract, with contributions from (**a**) photon and (**b**) beta radiation, as well as (**c**) the total dose.

**Figure 13:**
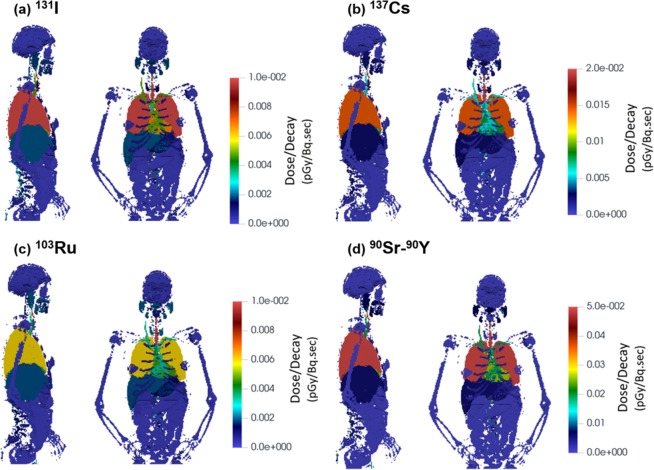
Dose to organs per decay from (**a**) ^131^I, (**b**) ^137^Cs, (c) ^103^Ru, and (**d**) ^90^Sr-^90^Y in the respiratory tract.

**Figure 14:**
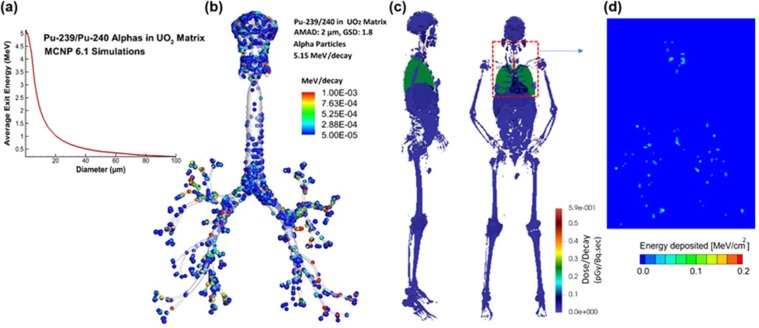
Dose to organs per decay for alpha particles from ^239^Pu or ^240^Pu: (**a**) average alpha-particle energy per decay as a function of UO_2_ aerosol diameter as calculated with MCNP, to account for self-shielding; (**b**) application of this distribution with the aerosol distribution in the respiratory tract to localize energy emitted per single decay in the body (MeV/decay), (**c**) dose to organs using source locations and average energies per aerosol in VIP-Man, and (**d**) average ^239,240^Pu alpha-particle energy emitted from UO_2_ aerosol based on aerosol size (projection image).

Deposited doses in different organs were calculated for ^131^I, ^134,137^Cs, ^90^Sr-^90^Y, ^103^Ru and ^239,240^ Pu per decay due to photons, beta particles, and alpha particles (Figs. 12, 13, 14). The doses are calculated as averages over each organ. Figure 12 shows the dose per decay in the organs for ^134^Cs, with Fig. 12a from photons, and Fig. 12b from beta particles. For both photon and beta, the trachea appears to have the highest dose among all organs (i.e., 59% higher than the lung). Due to the local energy deposition of beta particles, elevated doses were observed only in the trachea and lung in Fig. 12b. For photons (Fig. 12a), however, elevated doses were found not only in the trachea and lung, but also in the heart, liver, thyroid, and parts of the skull (Fig. 12a). In particular, a 14% higher dose of ^134^Cs photon energy dose in the heart was predicted than that in the lung. When considering the total dose (Fig. 12c), however, the lung receives a higher dose than the heart, because ^134^Cs beta particles deliver a 200% higher dose to the lung than to the heart.

The total organ dose per decay among four other radionuclides (^131^I, ^137^Cs, ^103^Ru, and ^90^Sr-^90^Y) is shown in Fig. 13. The total dose has considered both photons (gammas and x-rays) with the energy distribution given in Table 1 and betas with the spectra shown in Fig. 2. The exact values for the doses are shown in Table 3. In all cases, the trachea receives the highest dose. The heart to lung dose ratio is 0.50, 0.55, 0.77, 0.77, 0.40 for ^131^I, ^137^Cs, ^134^Cs, ^103^Ru, and ^90^Sr-^90^Y, respectively. This difference can be explained by the ratio of the photon energy emitted to that of beta decay. When that ratio is higher, the dose becomes less local, and as a result, more dose is delivered to peripheral organs such as the heart. The photon to beta energy ratio per decay is 2.35, 3.54, 12.1, 10.6, and 0 for ^131^I, ^137^Cs, ^134^Cs, ^103^Ru, and ^90^Sr-^90^Y, respectively. ^90^Sr doesn’t emit any photons. ^90^Y emits 2.186 MeV photons in 1.4E-6% of its decays and therefore is negligible compared to betas emitted every decay (Q value 2280 keV). On this ground, only betas were simulated for ^90^Sr and ^90^Y, which gives the lowest heart-to-lung dose ratio.

**Table 3:** Dose Deposition Distribution in Organs per Decay.

Organ Name	Mass (g)	Dose/Decay (pGy/Bq.sec)				
		I-131	Cs-137	Cs-134	Ru-103	Sr/Y-90
Bone-Articulations	895.1	8.88E-04	1.05E-03	1.98E-03	7.59E-04	2.99E-03
Bone-other	7196.3	1.68E-04	2.07E-04	5.61E-04	1.90E-04	2.85E-05
Nerve-Lateral Ventrical	6.0	1.03E-04	1.60E-04	4.49E-04	1.83E-04	1.50E-06
Nerve-Caudate Nucleus	9.3	1.03E-04	1.59E-04	4.44E-04	1.87E-04	4.57E-07
Nerve-Cerebellum	123.7	2.36E-04	3.51E-04	9.74E-04	4.14E-04	2.26E-06
Nerve-Gray Matter	682.3	9.27E-05	1.46E-04	4.09E-04	1.66E-04	8.26E-07
Nerve-White Matter	441.0	8.95E-05	1.41E-04	3.95E-04	1.61E-04	8.27E-07
Blood-Circulatory	853.2	4.78E-03	5.57E-03	1.00E-02	3.33E-03	1.89E-02
Nerve-Corpus Callosum	17.0	9.88E-05	1.53E-04	4.31E-04	1.79E-04	6.89E-07
Digestive	226.2	1.77E-03	2.04E-03	3.55E-03	1.95E-03	7.26E-03
Endocrine	1.9	1.78E-03	2.49E-03	6.80E-03	2.33E-03	1.99E-05
Esophagus Wall	39.9	3.97E-03	4.91E-03	1.07E-02	3.74E-03	6.72E-03
Eye	14.1	1.27E-04	1.97E-04	5.49E-04	2.38E-04	1.09E-06
Nerve-Lentiform Nuclus	13.8	1.16E-04	1.80E-04	4.98E-04	2.12E-04	8.24E-07
Bone-Mandinle	84.8	5.86E-04	7.59E-04	2.08E-03	1.00E-03	1.32E-05
Muscular	42963.9	1.89E-04	2.62E-04	6.88E-04	2.25E-04	1.41E-04
Nerve-other	245.7	4.33E-04	6.18E-04	1.70E-03	6.38E-04	4.67E-05
Testis	21.4	2.12E-06	4.73E-06	1.40E-05	3.06E-06	0.00E + 00
Nerve-Pons & Middle Cerebellar Peduncle	23.8	2.75E-04	4.06E-04	1.13E-03	4.91E-04	2.33E-06
Prostate	19.5	6.21E-06	1.21E-05	3.54E-05	8.91E-06	5.31E-09
Respiratory	15.5	2.49E-02	2.62E-02	3.26E-02	1.29E-02	1.29E-01
Skin	2296.2	6.78E-05	1.04E-04	2.88E-04	9.07E-05	5.85E-07
Bone-Cranium	804.4	1.51E-04	2.04E-04	5.67E-04	2.51E-04	2.39E-06
Bone-Spinal Bone	1107.1	2.20E-03	2.45E-03	4.32E-03	1.69E-03	8.08E-03
Upper Large Intestine	620.7	2.63E-04	3.77E-04	1.04E-03	3.30E-04	2.54E-06
Nerve-Thalamus	8.3	1.13E-04	1.75E-04	4.89E-04	2.06E-04	1.40E-06
Thyroid	27.5	1.35E-03	1.90E-03	5.22E-03	2.03E-03	1.53E-04
Fat	36728.2	3.97E-04	4.93E-04	1.02E-03	3.11E-04	1.22E-03
Lung	910.3	1.35E-02	1.43E-02	1.87E-02	6.01E-03	6.60E-02
Nerve-Fronix	1.5	1.22E-04	1.85E-04	5.14E-04	2.20E-04	8.83E-07
CSF-Skull CSF	91.8	8.10E-05	1.30E-04	3.63E-04	1.47E-04	6.30E-07
Stomach Wall	154.9	1.06E-03	1.45E-03	3.78E-03	1.37E-03	2.23E-03
Gall Bladder-wall	12.8	4.42E-04	6.28E-04	1.73E-03	5.88E-04	3.83E-06
Liver	1935.5	1.93E-03	2.37E-03	4.92E-03	1.88E-03	5.81E-03
Rectum	45.0	7.28E-06	1.40E-05	4.07E-05	1.00E-05	1.01E-07
Small Intestine	1236.2	1.43E-04	2.08E-04	5.76E-04	1.79E-04	1.21E-06
Kidney	337.9	2.14E-04	3.06E-04	8.50E-04	2.65E-04	1.87E-06
Pancreas	82.8	4.53E-04	6.34E-04	1.75E-03	5.74E-04	4.39E-06
Spleen	239.9	4.69E-04	6.54E-04	1.80E-03	5.52E-04	4.60E-06
Thymus	11.5	3.12E-03	4.49E-03	1.22E-02	4.15E-03	3.80E-03
Urinary Bladder-wall	40.5	8.63E-06	1.64E-05	4.70E-05	1.22E-05	5.89E-08
Heart Wall	401.8	6.71E-03	7.87E-03	1.44E-02	4.64E-03	2.62E-02
Adrenal	8.0	4.49E-04	6.31E-04	1.74E-03	5.56E-04	3.80E-06
Reproductive	14.0	6.90E-06	1.33E-05	3.84E-05	9.65E-06	1.30E-08
Urinary System-other	13.9	1.17E-04	1.72E-04	4.75E-04	1.47E-04	7.56E-07
CSF-Spinal CSF	168.2	1.60E-03	1.88E-03	3.59E-03	1.46E-03	4.04E-03
Stomach Content	326.6	2.09E-03	2.47E-03	4.77E-03	1.85E-03	6.24E-03
Bone-tooth	35.2	4.91E-04	6.52E-04	1.78E-03	8.93E-04	9.89E-06
Lower Large Intestine	373.6	1.80E-04	2.57E-04	7.09E-04	2.16E-04	1.59E-06
Gall Bladder-Content(Bile)	29.4	3.68E-04	5.24E-04	1.45E-03	4.91E-04	3.39E-06
Stomach Mucosa	13.6	1.08E-03	1.49E-03	3.95E-03	1.35E-03	1.44E-03
Len	1.1	1.22E-04	1.89E-04	5.26E-04	2.21E-04	1.29E-06
Nerve-Optic Chiasma	0.3	1.84E-04	2.74E-04	7.69E-04	3.42E-04	2.24E-06
Air-Inside	1.0	9.85E-05	1.48E-04	4.11E-04	1.44E-04	2.02E-06
Bone-Red Bone Marrow	1118.4	6.28E-04	8.21E-04	1.93E-03	7.04E-04	1.31E-03
Nerve-Vestibulocochlear	0.1	3.42E-04	4.90E-04	1.36E-03	6.15E-04	7.06E-07
Esophagus Lumen	26.6	2.35E-03	3.26E-03	8.71E-03	3.17E-03	1.11E-03
Esophagus Mucosa	3.3	2.28E-03	3.20E-03	8.73E-03	3.02E-03	1.24E-03
Nerve-Optic Nerve	1.7	1.49E-04	2.29E-04	6.36E-04	2.76E-04	2.10E-06
Urinary Bladder- Content	43.9	8.71E-06	1.61E-05	4.67E-05	1.23E-05	6.24E-08
Male Breast	33.6	4.04E-04	6.01E-04	1.66E-03	4.98E-04	3.19E-06
Absorbed Fraction (Photons)		0.46	0.44	0.44	0.45	—
Absorbed Fraction (Betas)		1	1	1	1	0.998

Figure 14 shows the dose distribution and 2D-projected energy deposition density of ^239,240^Pu from alpha particles. Two approaches were considered in order to account for aerosol self-shielding: (a) point source approach with volume-weighted average exit energy (b) volume source approach which directly simulated alpha shielding in UO_2_ in the same calculation. In the first approach, the dose per decay was calculated by first running MCNP shielding calculations (Fig. 14a) to determine the exit energy as a function of sphere diameter per decay. These simulations considered uniformly distributed plutonium source in UO_2_ spheres with different sizes at room temperature. The average exit energy decreases as a function of the diameter of the carrier aerosol. After that, the volume-weighted average exit energy was calculated for the deposited aerosol particles (2-µm AMAD and 1.8 GSD for ^239,240^Pu), as shown in Fig. 14b. Figure 14c shows the organ dose per decay from ^234,240^Pu alpha particles by considering all deposited particles from CFPD predictions. As expected, elevated doses were noted in the trachea and lung only since the mean free path for alphas in tissue is less than 50 microns. In this approach, the sources, however, have been defined as point sources with shielding accounted for by weighting the position of each source particle by the product of the exit energy for its diameter and its volume. The volume-weighted average exit energy was used for these simulations.

Figure 14d shows the spatial distribution of alpha radiations. To preserve position-energy data and reduce regression error, direct simulations of aerosol shielding were explored inside the VIP-Man model, rather than doing it separately. The direct simulations used volume sources rather than point sources and simulated the UO_2_ spheres in the same simulations inside the VIP man model. The hotspots of alphas energy deposition in Fig. 14d appear in the throat and at the bifurcation, while others are scattered throughout the lung parenchyma.

## Discussion

A new model was introduced that coupled CFPD aerosol transport code with MCNP6 to predict the energy deposition distribution due to radioactive aerosols decaying in the respiratory tract. Supplemental computer codes have been developed to visualize MCNP6 output, register source particles in MCNP6 based on CFD results, consider alveolar deposition, and calculate mass-based deposition fractions of polydisperse particles. One similar study has been reported by Kim *et al*.^88^, who coupled ANSYS Fluent with MCNP6 to analyze the design of a solution vessel based Mo-99 production facility. In the current study, we demonstrated the usage of the CFPD-MCNP methodology in a physiology realistic respiratory airway model and VIP-Man phantom. Radiation doses in 61 different organs were computed for ^131^I, ^137^Cs, ^134^Cs, ^103^Ru, ^90^Sr-^90^Y, ^239-240^Pu due to gamma radiation, x-rays, beta decay, and alpha decay of the radionuclides in the respiratory tract. Radionuclides were not tracked in dissolution and absorption in lung fluids and transfer through the body as this is beyond the immediate focus of the present study and is specific to the carrier aerosol particle. It is known that by translocation, ^131^I accumulates in the thyroid, and Pu appears in the liver and bones^89,90^. For radionuclides that are deposited on the airway surface and decaying there, the organs of high risk include the trachea, lungs, heart, thyroid, and liver. The trachea receives the highest dose, followed by the lung and the heart. For radionuclides with high gamma energies and low beta energies, the dose delivered in the heart can be very close to that of the lung. The heart to lung dose ratio is 0.50, 0.55, 0.77, 0.77, 0.40 for ^131^I, ^137^Cs, ^134^Cs, ^103^Ru, and ^90^Sr-^90^Y, respectively. The absorbed beta fraction in the entire body was 1.0 for all cases with the exception of ^90^Y where it was 0.998. For photons, the absorbed fraction decreased with the increase in photon energy but was in the range 0.44-0.46. This implies that ~55% of the photon radiation escapes the body to the environment, which can put other nearby individuals to risk.

A major advantage of this newly coupled CFPD-MCNP model is its ability to consider more realistic radiation exposure scenarios in the lungs. In this case, we looked at radiation dose from CFPD-predicted lung-based particles, but the lung deposition can be coupled with biokinetic models to expand dosimetry predictions to other organs. Because carrier aerosol properties, respiratory geometry, and breathing condition can be considered directly by the CFPD model, it is well suited to study radiation dosimetry and assess health risk for subjects under realistic environmental and occupational exposures. The International Commission on Radiological Protection (ICRP) has emphasized the importance of the consideration of uncertainties in assessing cancer probability for an individual and in estimating doses in epidemiological studies^63^. In this study, substantial differences were observed between the CFPD predictions and the MPPD results in both the head and TB region with oral breathing (Fig. 5). A much higher sensitivity of the CFPD model to the variations in flow rates and particle sizes was observed for both the total and regional deposition rates (Fig. 5). This sensitivity will be crucial when considering intersubjective dosimetry variability for a given group. This highlights the need to include the anatomical details of the respiratory tract for improved inhalation dosimetry. Also, detailed deposition mapping can be predicted using CFPD, while this information is absent in the MPPD or ICRP models. The knowledge of local particle deposition is essential for the health assessment of alpha emitters and has been demonstrated to play a key role in lung cancer development^91^.

The major reasons underlying the prediction differences between the CFPD and MPPD/ICRP models are the presentation of the respiratory tract geometry and its interaction with inhaled airflow and particles. CFPD considers the realistic respiratory tract *per se* with anatomical details, which are critical in determining flow dynamics (i.e., boundary layer development, vortex formation, turbulence generation/decay, etc.) and can substantially affect the behavior and fate of entrained particles. In contrast, MPPD/ICRP models replied on deposition data from well-developed tubular flows and were validated/improved with *in vitro* or *in vivo* dose coefficients^9,92^. Such models will be accurate within the experimental ranges that generated these dose coefficients but will become less reliable outside these ranges. The ICRP compartment model simplified the respiratory tract into 5 regions: ET1 (anterior nasal region), ET2 (main extrathoracic region), BB (bronchial), bb (bronchiolar), AI (alveolar interstitial region)^9^. In this sense, variations of the inhalation dosimetry due to airway geometrical details are neglected. In our previous studies, the geometrical complexity has been demonstrated to have significant impacts on the deposition rate of micrometer aerosols^67,93,94^ and the deposition distribution of nanoparticles^95^.

In this study, radiation doses were computed and compared in terms of different variables: photon fluence, energy deposition density, and absorbed dose. These variables are expressed as *per* radionuclide decay; in doing so, the results can be readily implemented to estimate cumulative doses for exposures of any duration with any aerosol concentration.

All gamma rays, x-rays, beta particles, and alpha particles emitted from a given radionuclide decay were considered in this study, with energies and intensities per decay as listed in Nudat2.7 tables [www.nndc.bnl.gov/nudat2]. As betas are emitted in a range of energies, the spectra were used, as shown in Fig. 2. Self-shielding within larger particles becomes important for alpha particles, which have high linear energy transfer, and so self-shielding was accounted for by either direct calculation with MCNP6 or adjusting down alpha energies emitted depending on the particle size, following MCNP6 simulations.

It is important to reiterate that this study is focused on CFPD simulations to model particle deposition in a physiologically realistic respiratory tract and combining that with MCNP6. The present work is only concerned with the phase after particle deposition and before diffusion to the blood. The internal radiation dosimetry was estimated from aerosol particles while they were still on the airway surface. The CFPD simulation results can be used as the starting point for follow-on simulations including internal dose evaluation after diffusion to the blood, which can be assessed using codes like LUDEP, IMBA, and BiDAS^2,58^.

Assumptions that may affect the realism of the results in this study include a rigid-wall airway geometry, a single and rigid phantom model, a limited number of sample aerosol particles, a limited number of MCNP simulation histories, and homogeneous composition at the organ level in the phantom. Previous CFPD studies have considered the influences from compliant airway walls and dynamic glottis^96–99^ Likewise, the 3-D VIP-Man is voxel-based and lacks the flexibility to deform or adjust posture^45,100^. More recently, since MCNP started supporting embedded Abaqus geometries, mesh-based phantoms appeared, which can deform and represent splines more accurately^101^. However, they suffered from a significantly longer pre-processing time and a much larger memory footprint. The computation time was 70–150 times longer than a comparable voxel-based phantom^102^. The VIP-Man phantom was based on an adult male of 186 cm in height and 103 kg in weight, which may not be representative of the human population. For polydisperse aerosols with lognormal distributions, a strictly count-independent particle may require billions of particles to eliminate the outliers’ (large particles) effects. It is also noted that the use of 100 million histories in MCNP6 simulations is inadequate to achieve satisfactory accuracy in regions distal from the source and receiving minor doses, such as the legs. However, 100 million histories’ simulation has been demonstrated accurate enough in the regions of interest both in this and other studies^59,60^.

In summary, detailed CFPD simulation and Monte Carlo radiation transport were coupled to model inhaled radioactive aerosols using a physiologically realistic lung model and a whole-body radiation phantom. Deposition fraction and distribution in various regions of the respiratory tract were simulated for different polydisperse particle size distributions representative of measured radioactive aerosols. Spatial distribution of energy deposition density was calculated for different radiation-emitting nuclides: ^131^I, ^134,137^Cs, ^90^Sr-^90^Y, ^103^Ru and ^239,240^Pu. Absorbed dose per decay was quantified for different organs and the whole body. Organs in the proximity of the respiratory tract, such as heart, arteries, thyroid, and liver, have high but different exposure levels and potentially biological risks. Compared to previous studies, the CFPD-MCNP model considered more realistic aerosol distribution, respiratory geometry, and respiratory condition, thereby it could provide more accurate radiation dosimetry estimations.

## Methods

### Study design

**Figure 15:**
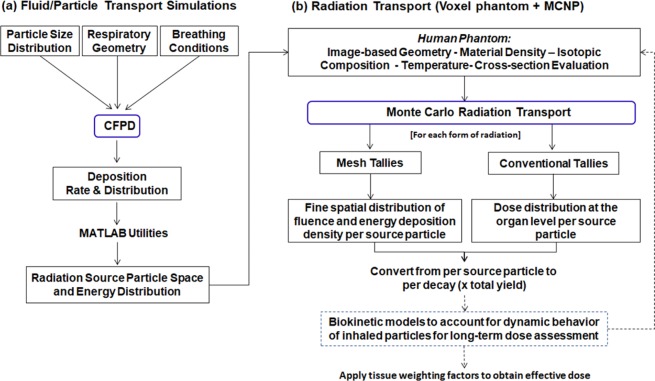
Flow chart of the multi-physics coupling methodology between CFPD and MCNP that consist of two steps: (**a**) fluid-particle transport simulations, (**b**) radiation transport simulation such as with VIP-Man phantom and MCNP.

The flow chart of the multi-physics coupling methodology between CFPD and MCNP6 is shown in Fig. 15. First, physiology-based simulations using CFPD were conducted to find the deposition rate and distribution of the radioactive aerosols based on realistic particle size distribution, respiratory geometry, and breathing conditions (Fig. 15a). Computer codes were written to convert aerosol particles to radiation source particles and to register these source particles to the lung of the VIP-Man phantom. Second, MCNP6 radiation transport simulations were performed to predict the spatial distribution of radiation fluence and energy deposition density per decay (Fig. 15b). Third, the radiation dose per decay due to gammas, x-rays, and betas was calculated for 61 organs defined in the VIP-Man phantom (Table 3) for ^131^I, ^134^Cs, ^137^Cs, and ^103^Ru. The dose due to betas from ^90^Sr-^90^Y was also calculated and that due to alphas in ^239,240^Pu while considering shielding of alphas by the carrier aerosol particle.

### Physiology-based simulation of aerosol deposition

A respiratory tract model that was previously developed in our laboratory was used in this study^103,104^. This model consists of the mouth-throat (MT) and a lung model with nine bifurcating generations (G9). The lung geometry was further divided into tracheobronchial (TB) region, the right lung (RL), and the left lung (LL), as shown in Fig. 3. The mouth-throat has been previously developed from CT images of an adult^94^ and contains the oral cavity, pharynx, and larynx. The lung model was reconstructed from an anatomical replica^105^ *The cartilage rings*, which prevents the airway walls from collapsing, were retained in the TB model from the trachea to the fourth bifurcating generation^106^. There are 115 outlets (bronchioles) in the lung model.

A particle was tallied as deposited at the point of contact with the respiratory tract material. To quantify the deposition rate and deposition distribution of respirable radioactive aerosols, both inhalation and exhalation were considered. The modeling was performed for a single inhalation and exhalation. There are three steps for each deposition test: (1) aerosols inhaled into the mouth were tracked through the mouth-lung geometry using CFPD during inhalation, (2) for aerosols exiting the mouth-lung geometry, deposition was considered based on acinar empirical correlations and an appropriate fraction of aerosol particles were removed, and (3) during inhalation, the remaining aerosol particles were injected into the distal bronchioles and tracked through the mouth-lung geometry using CFPD. The details of each step were provided as follows.

For all simulations, the initial aerosol particles have a log-normal size distribution, as specified by the AMAD and GSD for each radioactive isotope (Table 1). During inhalation, an inlet condition of 0.683 m/sec was specified, which is equivalent to a volume flow rate of 15L/min. A discrete phase model was employed to track the particles in the mouth-lung geometry. As much of the particles were in the nanometer regime, user-defined functions were used to account for Brownian motion, Stokes-Cunningham drag, and near-wall drag force. The particles were tracked until they deposited on the walls or escaped from the 115 distal bronchioles.

The aerosol particles that escaped the mouth-lung filtration during inhalation were considered entering the alveolar region. Both the position and velocity of these particles were recorded at one of the 155 distal bronchioles. These particles were subsequently separated into two groups by removing an appropriate fraction based on existing empirical correlations^92^. One group represented the aerosols deposited in the alveoli and remained at the distal bronchioles, and the other group was released into the lungs at the beginning of exhalation via the distal bronchioles by reversing their velocities. Considering that the particles entering the alveoli are not uniform in size and had different alveolar deposition rate^92^, particle removal was performed based on the particle’s diameter. A MATLAB utility was developed to read all particles entering the alveoli. For each particle, a biased coin was tossed to decide whether to remove the particle. The removal probability was the product of the size-dependent empirical alveolar deposition fraction and the ratio of injected particles over exiting particles in the same diameter bin. Multiplication by the aforementioned ratio is to correct for the fact that the empirical alveolar deposition fractions were based on the total inhaled particles at the mouth inlet^92^. Exhalation was simulated by specifying zero pressure at the mouth and 46.1 Pa at the lung bronchioles, which gave rise to an expiratory flow rate of ~15 L/min.

A well-tested low-Reynolds-number k-ω turbulence model was used to resolve the respiratory flows^94^. A discrete-phase Lagrangian-tracking model was implemented to track the particle trajectories. The particle transport equation is shown below^94^:

1 $$\frac{d{v}_{i}}{dt}=\frac{f}{{\tau }_{p}{C}_{c}}({u}_{i}-{v}_{i})+{g}_{i}(1-\alpha )+{f}_{i,Brownian}+{f}_{i,lift}$$

where *v*_*i*_ is the velocity of the particles, *u*_*i*_ is the velocity of the flow, *f* is the drag factor^107^, *τ*_*p*_ is the particle reaction time, and *C*_*c*_ is the Cunningham correction factor^108^. User-defined functions (UDFs) were applied to consider the near-wall effect^109^ and Brownian motion force^110^. This model has been demonstrated to agree with *in vitro* measurements in previous studies for both nanoparticles^110^ and micrometer particles^111–113^. ANSYS ICEM CFD (Ansys, Inc) was used to generate the computational mesh. A grid independence study was done by testing different mesh sizes and resultant deposition rate^114,115^. In the final mesh, there are 2 million cells, with the first near-wall cell having a height of 0.05 mm.

### Registration of CFPD deposition data with VIP-Man phantom

Once the 3D deposition distribution was obtained, aerosol particles needed to register with the lung space in the VIP-Man phantom for subsequent radiation dose modeling with MCNP6. This involves two processes: (1) registering the CFPD lung geometry with the VIP-Man lung phantom, and (2) converting the aerosol particles to radiation source particles. To these two aims, three utilities were developed. The first utility converts the VIP-Man phantom into the ParaView format to identify the scale factor of the lung model to overlap with the VIP-Man’s lungs. The second utility was developed to collectively rotate and translate the aerosol particles so that they fit into the space of the lung phantom. The Third utility, which is connected to the second, generates MCNP6 radiation source cards by converting aerosol particles into source particles with radionuclide specific discrete energy distribution in case of photons and energy spectrum in case of betas and updates the MCNP6 input file. As inhaled aerosol particles are not uniform in diameter, the photon source particles were duplicated by a factor proportional to the aerosol particle’s volume (or mass) for appropriate radiation scaling. This process required linearly binning the particles by volume and then multiplying the particles based on the volume of the bin they fell into. These were run for dosimetry from alpha, beta, electron, gamma-ray, and x-ray emission, which are then presented in a per-decay scaling.

Several radiation quanta are typically emitted in each radionuclide decay. All radiation emitted from each nuclide (gammas, x-rays, and betas) was simulated. Auger electrons were not considered as they have very low energy (usually <10 keV) and occur in a small fraction of decays as given in Nudat2.7 tables at the National Nuclear Data Center^116^. Gamma-ray and x-ray radiation can have a high likelihood of leaving the lungs and depositing energy in other organs. Within the lungs themselves, there is an absorbed fraction from photons, but charged particle emissions deposit most of their energy in the lungs and trachea, and as a result, they represent a large fraction of the local dose in the respiratory tract. Alpha particles have high stopping power and on average lose a fraction of their energy within the UO_2_ aerosol particles. This self-shielding in the particles was calculated for the intensity weighted average ^239^Pu and ^240^Pu alpha particle energy, 5.15 MeV in both cases, with the average alpha energy leaving the aerosol per decay presented as a function of particle diameter (Fig. 14a). The Pu was ejected in fuel particulates from the Chernobyl accident, estimated to be 1% ^235^U and 0.4% ^239^Pu by mass at the time of the accident^117^. With the much shorter half-lives of ^239^Pu and ^240^Pu than the U in the fuel, the specific activity of Pu is much higher, with 10^9^ Bq/g for those Pu isotopes compared with 10^4^ Bq/g for the U content. Even at 0.4% Pu, Pu activity is several orders of magnitude larger than U, and U is most important here as just a shielding material for Pu radioactivity.

For beta particles, using only the average energy overstates the locally absorbed fraction^76^, so full energy spectra were used in MCNP6 following spectra from Eckerman *et al*.^118^ and from Burrows^119^. The nuclide ^90^Sr is considered in equilibrium with its short-lived decay daughter ^90^Y, and the rates of decay of both are equal. Therefore, they are combined as a pair. ^90^Y emits betas with end point energy 2.28 MeV which can leave the lungs and deposit peripheral dose in neighboring organs. All other charged particle emission examined is fairly well contained within the lungs.

### MNCP6 code

MCNP6 is a stochastic method code, and it is critical to ensure that the resultant statistical error in the region of the interest is below a prespecified criterion. When calculating the radiation fluence, MCNP6 models the transport and interactions of each radiation source particle in the system by sampling the distance to a collision, then it probabilistically determines what interaction will take place (in the case of photons, for instance, this includes Compton absorption and scatter, and photoelectric absorption). In this study, each MCNP6 simulation for gamma rays was run for 100 million histories, beta particles were run with 20 million histories for fluence and, as for alphas, 100,000 histories for energy density and 20 million for dose^85,86^. The relative error is defined as the standard deviation of the mean of the tallied quantity divided by the mean itself and is proportional to the square root of the number of histories^120^. Independent simulations were carried out for different forms of radiation.

For average absorbed dose in organs from respiratory tract emissions, the F6 tally was used, averaging the MeV/g values in all voxels within each cell, with each cell corresponding with an organ, and finally converting to pGy/Bq-s. As MCNP normalizes the quantities per source particle, the tallies were multiplied by the total yield for the particular type of radiation to convert to per decay (i.e per Bq-s). The doses from different forms of radiation were added up and multiplied by 160.2 to convert from MeV/g/Bq-sec to pGy/Bq-sec. The organ doses from different forms of radiation were then added up to obtain the dose distribution per decay due to photons and electrons combined.

For high-resolution visualization of radiation fluence for photons (gammas and x-rays) and betas, the *fmesh* tally was used. The energy deposition density for photons was obtained by applying a tally modifier card to the fluence tally (*FM04 -1.0 0 -5 -6)*, which integrates the fluence with macroscopic cross-section for the material and the total photon heating per reaction along with the phase space in each mesh element. For alphas, a *tmesh* was used since *fmesh* doesn’t support alphas. Custom utilities were developed for visualization of the mesh tally, VIP man, and organ tallies in ParaView.

### Alpha particles

Given the very short mean free path of alpha particles in matter including tissue (<50 μm in water^121^) and the carrier particles, both the direct and indirect methods have been used to calculate the dose while accounting for shielding by UO_2_ aerosol particles. While the energy emitted per decay of ^239^Pu or ^240^Pu is ~5.15 MeV, the effective exit energy after accounting for shielding by the aerosol particles was 3.24 MeV. Since alphas don’t travel in tissue, the dose per decay may be directly calculated by dividing the effective exit energy per decay by the mass of the lung/trachea. This approach may be the simplest and most direct approach to calculate the average lung dose; however, obtaining spatially resolved information from this approach is difficult. One approach to obtain spatial information would be to directly use the deposition information from CFPD and assume that the alpha particles deposit their energy wherever they fall given that their mean free path in tissue is less than 50 μm. A probability distribution for the energy deposited could be obtained by multiplying the volume of each deposited particle by the average exit energy for its size and dividing by the total volume, as shown in Fig. 14b. However, it’s difficult to clearly visualize the dose distribution using this approach due to the large particle count. For this reason, MCNP coupling was also considered for alphas in order to obtain spatially resolved information.
